# A Review on LDH-Smart Functionalization of Anodic Films of Mg Alloys

**DOI:** 10.3390/nano11020536

**Published:** 2021-02-19

**Authors:** Mosab Kaseem, Karna Ramachandraiah, Shakhawat Hossain, Burak Dikici

**Affiliations:** 1 Department of Nanotechnology and Advanced Materials Engineering, Sejong University, Seoul 05006, Korea; 2 Department of Food Science and Biotechnology, College of Life Science, Sejong University, Seoul 05006, Korea; karna@sejong.ac.kr; 3 Department of Industrial and Production Engineering, Jashore University of Science and Technology, Jashore 7408, Bangladesh; shakhawat.ipe@just.edu.bd; 4 Department of Metallurgical and Materials Engineering, Ataturk University, Erzurum 25240, Turkey; burakdikici@atauni.edu.tr

**Keywords:** Mg alloys, anodic film, layered double hydroxide layer, smart functionalization, corrosion

## Abstract

This review presents an overview of the recent developments in the synthesis of layered double hydroxide (LDH) on the anodized films of Mg alloys prepared by either conventional anodizing or plasma electrolytic oxidation (PEO) and the applications of the formed composite ceramics as smart chloride traps in corrosive environments. In this work, the main fabrication approaches including co-precipitation, in situ hydrothermal, and an anion exchange reaction are outlined. The unique structure of LDH nanocontainers enables them to intercalate several corrosion inhibitors and release them when required under the action of corrosion-relevant triggers. The influences of different variables, such as type of cations, the concentration of salts, pH, and temperature, immersion time during the formation of LDH/anodic film composites, on the electrochemical response are also highlighted. The correlation between the dissolution rate of PEO coating and the growth rate of the LDH film was discussed. The challenges and future development strategies of LDH/anodic films are also highlighted in terms of industrial applications of these materials.

## 1. Introduction

Material degradation, specifically corrosion, is a serious issue limiting the use of active metals like magnesium in advanced applications [1]. Mg and its alloys have high specific strengths by which they can replace heavy metals in different technological sectors [1]. Therefore, it is highly important to improve the electrochemical stability of these materials in corrosive environments to extend their applications. To date, several methods, such as sol-gel coating, chemical vapor deposition, anodizing, and plasma electrolytic oxidation (PEO) have been utilized to enhance the protective properties of light metals and their alloys [2,3,4,5,6,7,8,9,10]. Among them, the anodizing method discovered in 1923 has been used extensively to form thin protective anodic films [7]. As an updated version of anodizing, PEO is an emerging method because of its unique plasma-in-water system. Typically, PEO transforms metal surfaces into a robust layer of their corresponding oxides using numerous micro-sized plasma discharges, which are generated as the result of electrical breakdown events at high overvoltages [7]. These micro-sized plasma discharges induce a high-temperature environment (T > ~3500 K) that is above the melting point of most metals and oxides [11], leading to a dynamic surface topography comprising micropores due to local and repetitive melting-solidification cycles. However, due to the thin layer and/or the porous structure of the anodic films produced via anodizing and PEO, the corrosive species would reach the metallic substrate, leading to its corrosion in extreme environments [7]. An additional treatment, therefore, must be applied to the anodic films towards achieving higher electrochemical stability for large-scale applications [7,12]. Several research groups have used several approaches, such as the typical sealing by boiling water [13], post-treatment using polymers or organic compounds [7,14], post-treatment by sol-gel coatings [15] in order to enhance the stability of anodic films of Mg alloys. However, the boiling water approach would not be desirable on account of the slight improvements in the protective properties as well as the high energy consumption associated with this method [7]. Moreover, the application of polymers and organic compounds to seal the anodic films would be limited due to the susceptibly of these materials to degradation at elevated temperatures. Also, the use of sol-gel coatings led to the formation of many cracks as a result of mismatching between the metal oxides incorporated by the sol-gel approach and MgO which is known to be the main component of the anodic films produced on Mg alloy via anodizing and PEO [7].

**Figure 1:**
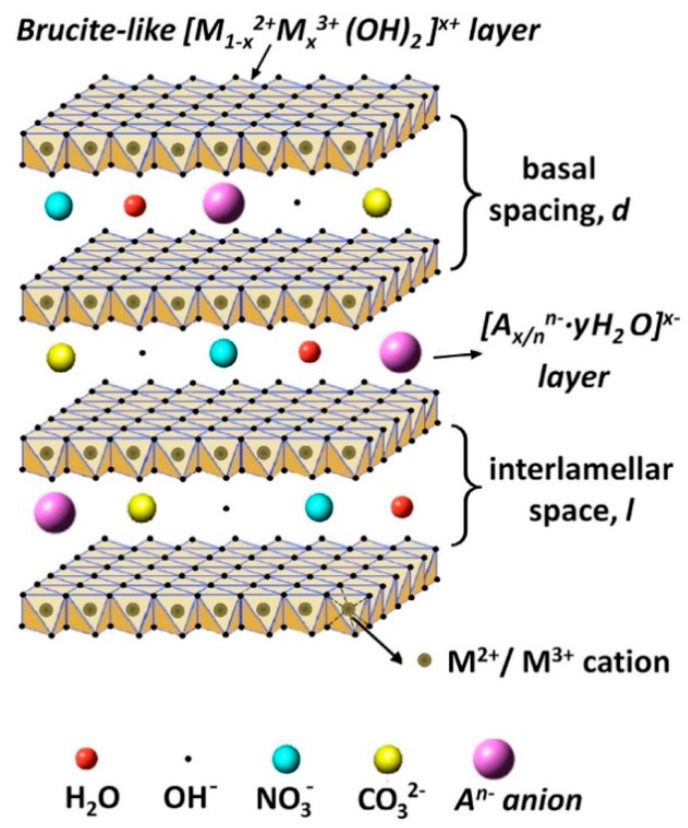
The general crystal structure of layered double hydroxide (LDH) film. Reprinted with permission from ref. [18]. Elsevier 2019.

In addition to the approaches described above, layered double hydroxides (LDHs) can provide another approach to improve the protective properties by increasing the barrier properties [16,17,18,19,20,21]. LDHs are lamellar crystals with positively charged brucite-like host layers with interlayer regions containing charge-compensating anions and solvation molecules [22,23]. The typical formula of these materials can be described as [M_1−x_^2+^M_x_^3+^(OH)_2_] (A^n−^)_x_/n·mH_2_O, where M^2+^ and M^3+^ are the divalent and trivalent cations, respectively, while, A^n−^ is the interlayer anion (Figure 1) [18]. Such inorganic nano-containers have been widely proposed to improve the corrosion resistance of Mg and its alloys on account of their merits, such as small size, high loading capacity, and simple modification [24,25,26,27,28,29,30,31,32,33,34,35]. Moreover, such materials have an excellent anion-exchange capability by the simultaneous release of interlayer anions and the adsorption of aggressive species from the corrosive environment. Therefore, LDH-based protective films can be considered as smart coatings, meaning that they can control the liberation of corrosion inhibitors and improve the long-term corrosion performance.

Anodization of the Mg alloys surface results in the formation of magnesium oxide (MgO) that acts as the major source of Mg^2+^ for the dense growth of LDHs, while LDH precursors are found to seal the anodic surface and can provide better active and passive corrosion protection with long-term stability. Moreover, the LDH films made on the anodic coating of Mg alloys can also increase the thickness of the protective film and, therefore, such factors could effectively stop corrosive species from reaching the metallic substrate. Thus, a review on the evolution of LDH materials made on the anodic films of Mg alloys focusing in depth on corrosion performance by covering the recent evaluation perspectives, trends in the synthesis methods, a deep insight into the mechanism, and the structure–corrosion correlation is urgently required. To the best of our knowledge, a review discussing the aforementioned aspects has not been undertaken. In this review, therefore, a short overview of the synthesis methods, formation mechanisms, and processing factors that affect the crystallinity and morphology of LDH films as well as the challenges and opportunities of developing advanced LDH-based materials with improved corrosion performance are elaborated coherently.

## 2. Synthesis of LDH Films on Anodized Mg Alloys

To date, several methods have been utilized to produce LDHs on the Al, Mg, and Ti alloy substrates, such as the co-deposition method [36,37], hydrothermal process [38,39], steam coatings method [40,41], electrodeposition [42], etc. These techniques were highlighted recently by Tabish et al. [43] and Guo et al. [44]. However, the methods that are usually employed to fabricate LDH films on the anodic films of Mg alloys are hydrothermal treatment and the co-precipitation method or a combination of both methods. Additional procedures, such as anion exchange reaction, and LDH reconstruction can be used to modify the LDH films to improve the protective properties of the LDH-based composites [43].

### 2.1. Co-Precipitation Method

Generally, LDHs can be fabricated by immersion of the anodic films of Mg alloys in a solution containing a selected ratio of divalent and trivalent metallic salts in the presence of the desired interlayer anion. Based on the type of metallic ions, the pH of the reaction medium during the synthesis process is usually controlled to be in the range of 7–11. However, several problems, such as the weak adhesion strength between the LDH film and the underlying substrate, complexity, time-consuming, low crystallization, and formation of large amounts of waste would be the main drawbacks of the co-precipitation method [43].

### 2.2. In Situ Hydrothermal Treatment

This feasible method has been employed in many studies to prepare homogenous LDH films on the anodic films of Mg alloys. Briefly, LDHs film can be obtained by immersing the anodic film in an aqueous solution containing NO_3_^−^ anions followed by hydrothermal treatment in a Teflon-lined autoclave at temperatures over 383 K. It is important to point out that autoclave conditions would limit the industrial applications of these materials, in particular the transport applications. Moreover, it is worth mentioning that the absence of autoclave conditions leads to the development of LDH films in carbonated electrolytes and the CO_2_-containing environment owing to the high sorption ability of LDH towards CO_2_ [45,46,47,48]. This led to the formation of so-called “dead” LDH film in which the intercalation of corrosion inhibitors became very difficult owing to the high charge density of CO_3_^2−^ anions, reducing the smart protection property of the film [49].

### 2.3. Anion Exchange

The LDH films are usually subjected to anion-exchange reactions to intercalate new anions into the gallery of LDH films. Therefore, it can be considered as an indirect approach to modify the structure and composition of LDH films. Anions of corrosion inhibitors, such as vanadate (VO_3_^−^) [50], and molybdate (MoO_4_^2^^−^) [51] are usually intercalated into the LDH films formed on the anodic films of Mg alloys. The LDH films intercalated with corrosion inhibitors would have a dual function: (i) entrapment of corrosive species and (ii) a controlled liberation of corrosion inhibitors. To sum up, although significant advances are achieved in the fabrication of LDH/anodic film composites of Mg alloys, two main challenges should be considered. First, how to increase the low adhesion strength between LDH coating and anodic films. Second, the formation of LDH films that occurs usually under autoclave conditions would significantly limit the industrial applications of these materials.

## 3. Transformation of Anodic Film to Layered Double Hydroxide (LDH) Film

To date, two hypotheses have been proposed to describe the formation mechanism of LDH films. First, Mg(OH)_2_ would play a precursor role in LDH formation [52,53]. As reported earlier [52], the Mg(OH)_2_ was deposited with a layered structure, while Al(OH)_3_ caused amorphous filamentary agglomerates during the initial formation stage. Then, the tetrahedral coordination of Al atoms will be transformed into octahedral ones, which are features of the LDH film. Second, the precursor of LDHs is Al(OH)_3_. Here, the amorphous colloidal Al(OH)_3_ was generated at the initial formation stage of LDH and then converted into crystallites of aluminum oxide hydroxide [54]. Besides the continuous incorporation of surrounding Mg^2+^ and CO_3_^2^^−^ ions, the integrated plate-like structure of LDH was obtained. In both hypotheses, however, the co-precipitation of Al(OH)_3_ and Mg(OH)_2_ would take place without the generation of polynuclear hydroxo complexes, and precipitates containing either Al(OH)_3_ or Mg(OH)_2_ were formed at the initial stage [52].

**Figure 2:**
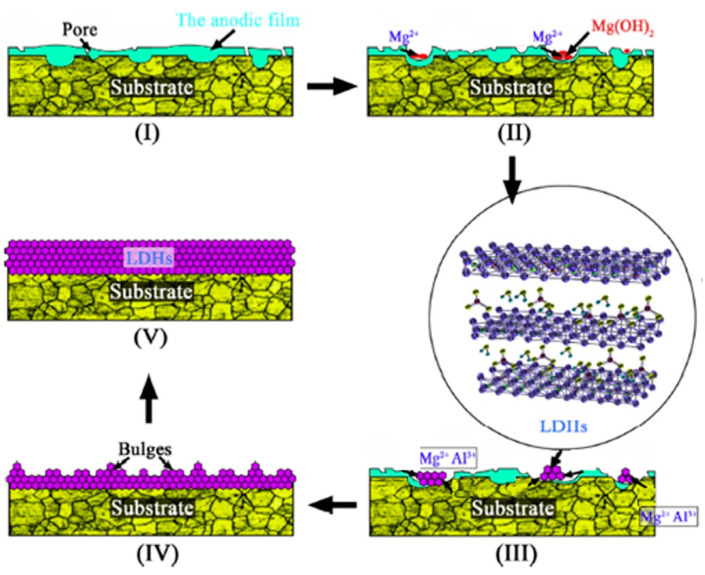
Schematic diagram of the transformation process of anodized film into LDH film. Reprinted with permission from ref. [55]. Copyright 2018 Elsevier.

As postulated by Zhang et al. [55], the internal sources of Mg^2+^ and Al^3+^ cations required for the development of LDH films were attributed to the dissolution of both the anodic films and AZ31 Mg alloy substrate. Here, the AZ31 Mg alloy samples were anodized in an electrolytic solution comprising NaAlO_2_ and NaOH for 30 min at a voltage of 20 V. The hydrothermal treatment of the anodized film in a solution of 0.1 M NaNO_3_ at 398 K in an autoclave led to the formation of LDH film. Based on the author’s findings, a Stranski–Krastanov (SK) two-dimensional layer growth (2D) to three-dimensional (3D) growth mode was suitable to elucidate the transformation from the anodic film to the Mg–Al LDHs film [55]. Figure 2 demonstrates the transformation process of the anodic film into LDH film. (I) An anodic film containing MgAl_2_O_4_, amorphous MgO, and amorphous Mg(OH)_2_ can be made on the surface of AZ31 Mg alloy as a result of the anodizing process in the alkaline-aluminate electrolyte. (II) A layered structure tended to be formed by the gradual gathering of the amorphous Mg(OH)_2_. Mg^2+^ cations resulting from the dissolution of the anodic film would combine with OH^−^ anions, forming sphere-like aggregates Mg(OH)_2_ with a layered structure [56]. This would lead to covering the micropores of the anodic film, resulting in the formation of a smooth surface. (III) The AZ31 Mg alloy substrate would also dissolve upon increasing the hydrothermal treatment time which resulted in a considerable increase in pH of the hydrothermal solution. The main reactions that happened during the LDH film formation can be summarized as follows:

(1) $$\mathrm{Mg}+H^{+}+H_{2}O\to {\mathrm{Mg}}^{2+}+{\mathrm{OH}}^{-}+H_{2}↑$$

(2) $${\mathrm{Mg}}^{2+}+2{\mathrm{OH}}^{-}\to \mathrm{Mg}{\left(\mathrm{OH}\right)}_{2}↓$$

Here, inwards growth of the LDHs film would happen as a result of the dissolution of AZ31 Mg alloy substrate and formation of Mg(OH)_2_. At the same time, Al^3+^ cations tended to incorporate into Mg(OH)_2_ structure which caused an imbalance in the sheet structure and the breaking down of the hydrogen bonds [54]. Thus, NO_3_^−^ inions tended to intercalate between the layers to maintain charge balance. (IV) At this point, LDH film grows inwards to the substrate and outwards to the film/solution interface simultaneously. As for the outwards growth, the LDH film would cover the entire surface accompanied by the formation of some sphere-like structures. Since the formation of islands would lower the strain energy in the crystals, their formation was energetically favorable. Thus, the growth model was transformed from 2D to 3D growth mode. (V) The crystallinity and thickness of LDH film tended to increase with time until the complete depletion of Mg^2+^ and Al^3+^ cations in the solution. Finally, it is worthwhile pointing out that the novel method of preparing Mg–Al LDHs described above has been also applied to describe the growth of LDH films on PEO coatings and to study the effect of PEO properties on the growth of LDHs [57,58].

## 4. Corrosion Performance of LDH/Anodic Film Composites

Growing LDH films on the surface of anodic films obtained by either anodizing or the PEO process would have several advantages as compared to the LDHs film fabricated directly on the Mg alloy substrate. First, the LDH film can adhere well to the anodic film. Second, the sealing of structural defects in the anodic film by the LDH films would provide a strong physical layer against the corrosive anions attack. Third, the intercalation of LDH film with corrosion inhibitors anions, such as MoO_4_^2−^ and VO_3_^−^ would lead to the development of smart self-healing films with excellent protective properties.

### 4.1. Conventional Anodizing

**Figure 3:**
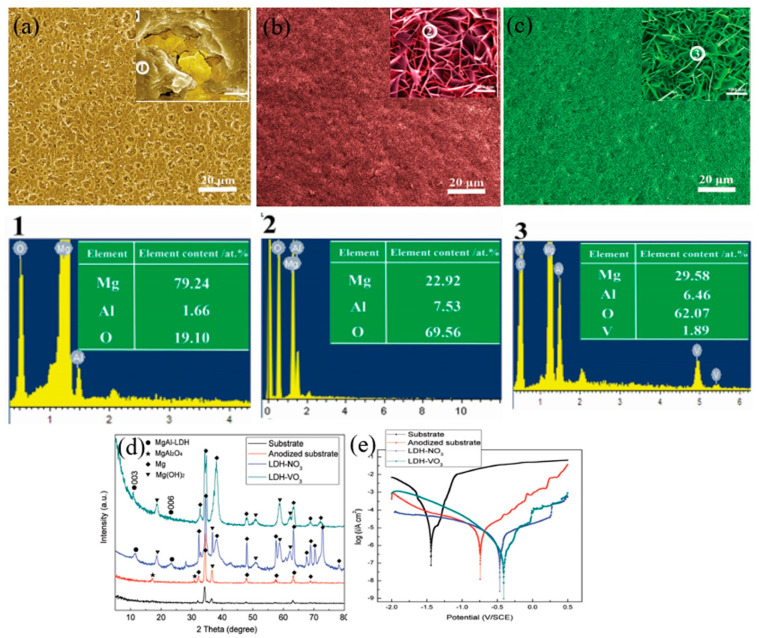
(**a**–**c**) Scanning electron microscopy (SEM) images showing the surface morphologies and the corresponding energy-dispersive X-ray spectroscopy (EDS) analysis of three different positions (1, 2, and 3) of the anodized sample, LDH-NO_3_ film, and LDH-VO_3_ film, receptively. The insets (scale bare = 500 nm) in Figure 3a–c represent the corresponding high-magnification images. (**d**) X-ray diffraction (XRD) patterns of the substrate, anodized sample, LDH-NO_3_ film, and LDH-VO_3_ film. (**e**) Potentiodynamic polarization (PPD) curves of the substrate, anodized sample, LDH-NO_3_ film, and LDH-VO_3_ film in 3.5 wt.% NaCl solution. Adapted with permission from ref. [50]. Copyright 2017 John Wiley and Sons.

LDH films are usually made on the anodized films of AZ31 and AZ91 Mg alloys that are generated by anodizing a solution containing NaOH and NaAlO_2_ at a voltage below 80 V. Here, the anodic films would supply sufficient Mg and Al mixed oxides to fabricate LDH films. Briefly, the anodic film obtained by the anodization of Mg alloy substrate in a suitable electrolyte could be exploited as internal sources of M^2+^ and M^3+^ cations which eliminate the need to add extra metal salts. Such a procedure would lead to the development of dense and uniform LDH film with good corrosion protection properties [59]. Following this approach, Zhang et al. [50] prepared a mixed oxide of Mg and Al through the anodizing of AZ31 Mg alloy in a solution comprising NaOH and NaAlO_2_. The voltage and anodizing time were controlled to be 20 V and 30 min, respectively. Such an anodic film would be the source of cations required for the preparation Mg–Al LDHs film. In other words, there would be no need to add any trivalent metallic salts during the synthesis of LDH film. Also, VO_3_^−^ anions were loaded into the LDH film through an anion-exchange reaction in a solution containing 0.1 M NH_4_VO_3_. Here, LDH-NO_3_ describes the LDH film produced on the anodized film of AZ31 Mg alloy while LDH-VO_3_ film refers to the LDH film subjected to an ion-exchange reaction. As shown in Figure 3a–c, the formation of LDH-NO_3_ and LDH-VO_3_ films led to an increase in the uniformity of the anodized film by blocking the micropores where a considerable change in the morphology of the anodized film was observed after the sealing treatment by LDH films (insets in Figure 3a–c). According to energy-dispersive X-ray spectroscopy (EDS) analysis performed at three different positions (1, 2, and 3), Mg, Al, and O elements were identified in all films while V element was detected only in the LDH-VO_3_ film due to the ion-exchange reaction. The formation of LDH-NO_3_ film on the surface of the anodized film was confirmed by the X-ray diffraction (XRD) patterns where two reflections of (003) and (006) were identified at 11.28° and 23.46°, respectively (Figure 3d). After the formation of LDH-VO_3_ film by an anion-exchange reaction, the two characteristic reflections of (003) and (006) were shifted to lower angles of 10.90° and 22.96°, respectively, suggesting the intercalation of the VO_3_^−^ anions into the interlayer galleries of LDH. Figure 3e shows the potentiodynamic polarization (PDP) of the samples in a 3.5 wt.% NaCl solution. The results of PDP tests showed that the values of *E_corr_* of AZ31 Mg substrate, anodized substrate, LDH-NO_3_ film, and LDH-VO_3_ film were −1.45 V, −0.74 V, −0.47, and −0.40 V vs. saturated calomel electrode (SCE), respectively while the corresponding corrosion current density (*i_corr_*) were 1.23 × 10^−5^, 3.89 × 10^−6^, 9.48 × 10^−7^, and 2.48 × 10^−7^ A·cm^−2^, respectively. As a result, LDH-VO_3_ film exhibited the lowest corrosion rate as this film had the smallest value of *i_corr_* and the noblest value of corrosion potential (*E_corr_*). This result was confirmed by the stable morphology of LDH-VO_3_ film after 144 h of immersion in 3.5 wt.% NaCl solution. The sealing effects originated from the disposition of LDH flakes and vanadium-rich oxide on the porous surface of the anodized film would be responsible for such improvements in corrosion resistance [50].

Wu et al. [60] studied the corrosion performance of superhydrophobic films on the anodized AZ31 Mg alloy modified with 1H, 1H, 2H, 2H-perfluorodecyltrimethoxysilane (C_10_H_4_Cl_3_F_17_Si) (PFDTMS), stearic acid (SA), sodium laurate (SL), and myristic acid (MA), respectively. The results demonstrated that the modification by PFDTMS could produce a better barrier to improve the corrosion resistance of AZ31 Mg alloy than the fatty acid with long carbon chains due to the formation of the Si–O-surface bond by a condensation polymerization reaction. Similarly, the in situ deposition of Mg–Al LDHs film on the anodized AZ31 Mg alloy followed by a modification by either MA or PFDTMS was examined by Zhang and co-workers [61]. LDHs with the biggest flakes were found to be the favorite adsorption sites for MA, while unfavorable for the adsorption of PFDTMS. The corrosion assessments in a 3.5 wt.% NaCl solution revealed that the super-hydrophobic Mg–Al LDH films obtained by intercalating MA showed higher corrosion resistance than the counterparts modified by PFFDTMS.

Very recently, Wu and coworkers [62] prepared a Ce-doped Mg–Al LDH film on the surface of anodized AZ31 Mg alloy by a hydrothermal treatment while VO_3_^−^ anions were loaded to LDH film via an ion-exchange reaction. The anodized samples were placed in a solution consisted of Al(NO_3_)_3_·6H_2_O, Ce(NO_3_)_3_·6H_2_O, and NH_4_NO_3_. The immersion time, temperature, and pH were controlled to be 12 h, 398 K, and 10.8, respectively. To intercalate VO_3_^−^ anions, the samples immersed in the above solution were immersed additionally in the solution of 0.1 M NaVO_3_ for 2 h at 45 °C at a pH of 8.4. The results indicated that a uniform and compact LDH film was obtained and the intercalation of Ce^3+^ and VO_3_^−^ ions would change the crystal structure of LDHs. As a result of the loading of Ce and VO_3_^−^ ions into the LDH film, the corrosion of the film was greatly suppressed, suggesting the co-intercalation of corrosion inhibitors into LDH film provided an effective method to protect AZ31 Mg alloy from corrosion attack.

**Figure 4:**
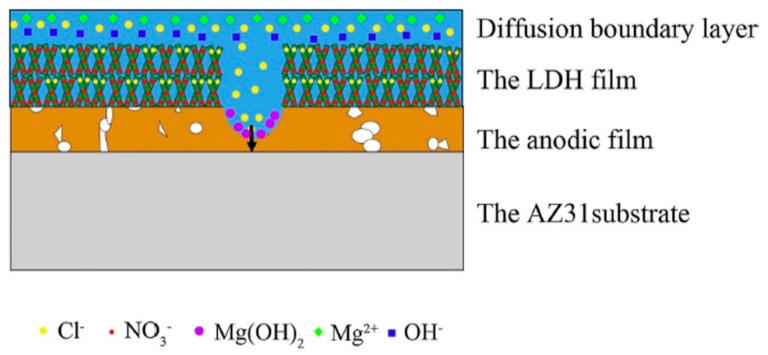
The proposed corrosion protection mode for the LDH/anodic film composite. Reprinted with permission from ref. [63]. Copyright 2018 Elsevier.

Wu et al. and co-workers in other works [50,55,63] successfully sealed the anodized AZ31 Mg alloy by LDHs films. AZ31 Mg alloy samples were oxidized in a solution of 7.14 g/L NaOH and 4 g/L NaAlO_2_ for 30 min at a voltage of 20 V. Afterward, Mg–M (M = Al, Cr, and Fe) LDH films were fabricated by placing the anodized samples in an aqueous solution composed of 0.05 M M(NO_3_)_3_ and 0.3 M NH_4_NO_3_ at 398 K for 12 h. The results indicated that the porous anodized film was completely sealed by LDH flakes. The electrochemical impedance spectroscopy (EIS) results revealed that the corrosion resistance ranked as follows: Mg–Al LDH > Mg–Fe LDH > Mg–Cr LDH > anodized AZ31 > AZ31substrate [63]. Two factors were responsible for the enhancement of protective properties of the anodized samples after the formation of LDH films. Firstly, the LDH films would completely seal the structural defects of the anodic film which hindered the corrosive species from reaching the substrate. Secondly, the LDH film as a nano-container has an excellent ion-exchange capability which enables the controlled liberation of corrosion inhibitors. Two such factors are summarized in the corrosion protection mechanism illustrated in Figure 4. In the proposed corrosion protection mechanism, four layers from top to the bottom, such as the diffusion boundary layer, the LDH film, the anodic film, and the AZ31 substrate, can be identified. Thus, the improved protective properties would be attributed to the ion-exchange capability, deposition of Mg(OH)_2_, and synergistic protection with anodic film.

As stated by Zhang et al. [55], a uniform and smooth surface could be formed on the surface of the anodized AZ31 Mg alloy as a result of increasing the hydrothermal treatment time from 0.5 h to 12 h during the synthesis process of LDH film. The compositional analysis results confirmed the formation of the LDH films. It was found that the morphologies of the LDH films formed on anodized AZ31 Mg alloys were found to be uniform and smooth. Consequently, the protective properties were significantly improved as approved by EIS and PDP measurements in a 3.5 wt.% NaCl solution.

The mechanism of the in situ formation of LDH films on the anodized film of AZ31 Mg alloy was proposed by Wu et al. [64]. The substrate was first anodized in an alkaline-aluminate solution, resulting in the formation of a porous anodic film, on which the LDH nuclei were directly produced. Under specific conditions, the nuclei gradually grew up, and then altered into the nanosheets, which assembled to make the LDH films. Such a conversion was greatly affected by the pH of the solution which played a crucial role in the generation of the LDHs film. The films obtained at the pH of 12.04 showed lower crystallinity of LDH nuclei and lower generation of the Mg–Al LDH films because the excessive OH^−^ would obstruct the reaction process from Mg^+^ to Mg^2+^, leading to a significant decrease in Mg^2+^ concentration which, in turn, restricts the generation of Mg_5_(CO_3_)_4_(OH)_2_·5H_2_O. As a result, the pores remain on the anodized sample, leading to poor corrosion resistance. In contrast, the films made in the pH between 10.72 and 11.72 exhibited a higher crystallinity of LDH flakes which resulted in high densification of the LDH structure. Such LDH films, therefore, could successfully seal the structural defects of the anodic films and reduce the corrosion rate of the AZ31 Mg alloy substrate.

In another work, Wu et al. [65] examined the correlation between the reaction temperature and the growth of Mg–Al LDH film fabricated on the anodized AZ31 Mg alloy via the in situ hydrothermal treatment method. Four different temperatures, such as 378, 388, 398, 408, and 418 K were used during the hydrothermal treatment. When T = 378 K and 388 K, the holes of the films of samples are larger and obvious; while T = 398 K, there are just some bumps on the surface; when T = 408 K and 418 K, the bumps were observed obviously from the surface of samples. The result revealed that the crystallinity of LDHs tended to improve with an increase in the reaction temperature. The results of the PDP tests in the corrosive medium implied that the values of *E_corr_* of LDH-378 K, LDH-388 K, LDH-398 K, LDH-408 K, and LDH-418 K were −1.278 V, −0.807 V, −0.701 V, −0.588 V, and −0.318 V vs. SCE, respectively while the corresponding *i_corr_* were 19.36, 1.459, 2.157, 2.199, and 1.036 µA·cm^−2^, respectively. Consequently, the LDH film obtained at 418 K had a higher corrosion resistance than other LDHs films. Using a solution composed of NaNO_3_ with 0.004, 0.032, and 0.256 M of Al(NO_3_)_3_, the same research group successfully in situ deposited Mg–Al LDH films on the anodized AZ31 Mg alloy samples [66]. The anodized AZ31 Mg alloy samples were immersed in the above solution (pH = 9) for 12 h at 398 K. The results indicated that LDH films obtained with a 0.032 M Al(NO_3_)_3_ solution had good crystallinity with higher impedance value, providing efficient protection for the anodized AZ31 Mg alloy substrate.

**Figure 5:**
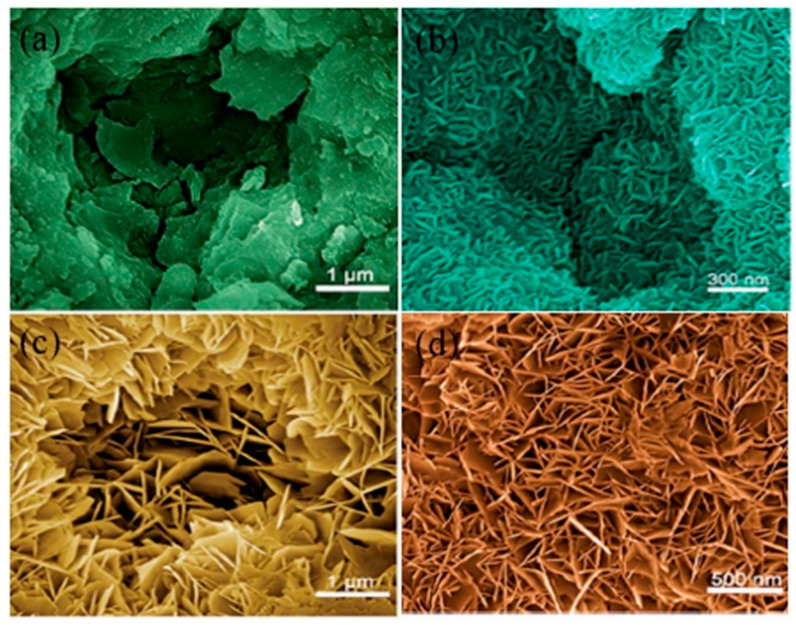
(**a**–**d**) SEM images showing the surface morphologies of the “A” (anodized film), “As” (anodized film sealed by boiling water), “A-LDH” (LDH film formed directly on the anodized film), and “AS-LDH” (LDH film formed on the sealed anodized film), respectively [67].

A novel method without adding extra salts during the formation of LDH film was proposed by Zhang et al. [67] who examined the influence of the sealing by boiling water on the growth and corrosion resistance of Mg–Al LDH film made on anodized AZ31 Mg alloy. For this reason, the anodized AZ31 Mg alloy samples were first treated by boiling water for 20 min and then by hydrothermal treatment in distilled water at 398 K for 12 h under autoclave conditions. Here, “A” refers to the anodized film, “AS” refers to the anodized film sealed by boiling water, “A-LDH” describes the LDH film formed directly on the anodized film, while “AS-LDH” indicates the LDH film formed on the sealed anodized film. The results presented in Figure 5a–d showed that the porous anodized film tended to be sealed by the LDH film where a fine and compact LDH film was formed when the anodized sample was sealed by boiling water before the sealing by LDH. This result would be ascribed to the fact that the anodized film during the sealing treatment by boiling water would have high kinetic energy and reactivity. Accordingly, the amorphous Mg(OH)_2_ gradually gathers in blocks to form the LDH structure. The presence of a high nuclei density in a given area would prevent the growth of individual particles in 2 dimensions, leading to the development of a film layer comprising numerous Mg(OH)_2_ crystallites in the nano-size range. Owing to the fine and compact morphology of the LDHs film made on the sealed anodized film, therefore, the AS-LDH sample exhibited a superior corrosion resistance in comparison to other samples.

### 4.2. Plasma Electrolytic Oxidation (PEO) Coating

In general, PEO may provide several advantages over anodizing, such as higher adhesion strength, high hardness, higher thickness, as well as the formation of crystalline phases under plasma conditions [7,12,68]. However, the structural defects formed inevitably during the PEO process would facilitate the inward motion of the corrosive medium into the substrate. In this regard, the sealing of such structural defects by the inclusion of nano-particles to the electrolyte during PEO or by using the post-treatments by boiling water, organic compounds, and sol-gel films have been proposed by several research groups [67,68,69,70,71,72,73,74,75,76,77]. Similar to the anodized samples, the successful formation of the LDHs on the PEO-coated samples not only caused an effective sealing of the structural defects but also released the corrosion inhibitors, providing the function of self-healing properties [19,43,78,79,80,81,82,83].

#### 4.2.1. Correlation between the Dissolution Rate of PEO and Growth of LDH

The elevated temperatures associated with the formation of PEO–LDH composite coatings would lead to a mismatch between the dissolution of PEO layers and the growth of Mg–Al LDH film [84,85,86,87,88,89]. This deficiency would restrict the large improvement in the protective properties of these materials. To fabricate a composite coating with superior corrosion performance, therefore, the dissolution rate of the PEO coating must be far slower than the growth rate of the LDH film. Thus, it is challenging and difficult to discover the equilibrium point between the dissolution rate of the PEO coating and the growth rate of the LDH film. Here, three cases can be discussed based on the dissolution rate of PEO coating and LDH film. First, if the dissolution rate of the PEO coating is greater than the growth rate of the LDH film, the protective properties of the composite coating may be limited. For instance, Chen et al. [84] used a hydrothermal treatment method in a 0.1 M Al(NO_3_)_3_ and 0.6 M NH_4_NO_3_ solution at 368 K with a pH of 7 for 1 h to prepare Mg–AL LDH on the PEO coating of AZ31 Mg alloy. The results revealed that a low pH value resulted in cracking in the PEO coating. The *i_corr_* of the composite coating instead increased one order of magnitude that of the PEO coating, which demonstrated that the corrosion resistance of the hybrid coating cannot reach the as-expected target. The authors also prepared different LDH films, such as Zn–Mg LDH, Ni–Mg LDH films, and compared the corrosion results with the Mg–Al LDH film and single PEO coating. They claimed that the type of cation loading LDH film played a key role in the protective properties of the composites. The Ni–Mg LDH film showed the highest corrosion resistance in comparison to other films because the porous structure of the PEO coating was completely sealed by the flakes of this film. In contrast, the Zn–Mg LDH film showed inferior improvement in the corrosion resistance as only a small number of micropores of original PEO coating were sealed by this film. Second, if the dissolution rate of the PEO coating is identical to the growth rate of the LDH film, this may cause a certain improvement in the protective properties due to the partial sealing of structural defects of PEO coating by LDH films. As an example, Peng et al. [85] made a Mg–Al LDH film on the PEO-coated AZ31 Mg alloy by hydrothermal treatment at 293 K for 12 h using a solution with a pH of 12.8. PDP results in a phosphate-buffered saline solution indicated that the *i_corr_* of the PEO coating was only reduced from 9.45 × 10^−6^ A cm^−2^ to 3.92 × 10^−6^ A cm^−2^ upon the application of Mg–Al LDH film on the surface of PEO coating, suggesting a slight improvement in the corrosion resistance was achieved. Moreover, the cytocompatibility tests using rat bone marrow stem cells indicated that the PEO–LDH composite can be applied as a promising implant in orthopedic surgery as it has better cytocompatibility property in comparison to the single PEO coating.

Third, if the dissolution rate of the PEO coating is less than the growth rate of the LDH film, the protective properties of the composite coating will be enhanced. For instance, Jiang et al. [86] fabricated a Mg–Al LDH film on the PEO coating of AZ91 Mg alloy by co-precipitation and hydrothermal treatment for different periods, such as 8, 20, 36, 48 h at 393 K. The hydrothermal solution was composed of appropriate concentrations of Mg(NO_3_)_2_·6H_2_O, Al(NO_3_)_3_·9H_2_O, and Na_2_MoO_4_·2H_2_O. The findings suggested that the *i_corr_* decreased from 1.27 × 10^−6^ A cm^−2^ for PEO coating to 1.03 × 10^−7^ A cm^−2^ for PEO-LDH film, which shows an increased corrosion resistance. Moreover, a self-healing capability with active inhibition ability of AZ91 Mg alloy was observed through the modification of the LDH film loaded with MoO_4_^2−^ anions by fluoroalkyl silane and infusion of perfluoropolyether treatments.

Chen et al. [87] successfully grew Mg–Fe LDH film on the surface of PEO coating of AZ31 Mg alloy via hydrothermal treatment in a solution containing appropriate concentrations of Fe(NO_3_)_3_·9H_2_O and NH_4_VO_3_ at 398 K for 24 h using an autoclave reactor. They found that the structural defects of PEO coating would act as ideal sites for the growth of LDHs flakes. Since a slight decrease in the thickness of the PEO coating was observed after the formation of LDH film, the authors postulated that the formation of strong adhesion between the PEO and LDH film would be strongly affected by the dissolution rate of PEO coating. However, the PEO/LDHs composite coating showed better corrosion resistance as compared to the single PEO coating.

According to Li and co-workers [88], an extremely thin Mg(OH)_2_ film was prepared using in situ growth on PEO-coated Mg alloy AZ31. It is worth noting that it has a lower preparation temperature at 333 K and a higher pH of 13.60. The composite coating has good corrosion resistance, and its *i_corr_* decreased three orders of magnitude than that of the substrate. This result implied that the growth rate of the Mg(OH)_2_ was greater than the dissolution rate of the PEO coating.

**Figure 6:**
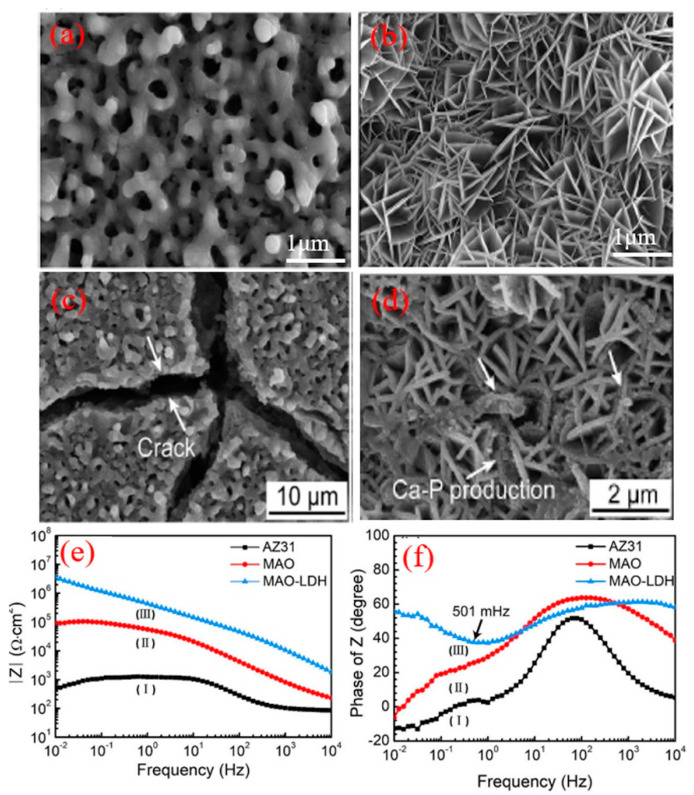
SEM images of the plasma electrolytic oxidation (PEO) coating and PEO–LDH composite coating before and after immersion for 154 h in 3.5 wt.% NaCl solution where (**a**–**c**) PEO coating and (**b**,**d**) PEO–LDH coating. (**e**,**f**) represents the Bode plots of AZ31 Mg alloy substrate, PEO coating, and PEO–LDH coating, respectively [89].

Following this approach, the same research group [89] suggested that the synthesis of LDH film on the surface of PEO-coated AZ31 Mg alloy can be successfully performed when the PEO coating was immersed in a solution containing EDTA-2Na and NaOH for 48 h at a relatively low temperature of 333 K which led to slow down the dissolution rate of PEO coating in comparison to the growth rate of the LDH film. As compared to the morphology of the PEO coating and PEO/LDH composite coating shown in Figure 6a,b, the PEO–LDH composite coating possessed self-healing ability whereby no cracks are observed on the surface of the composite coating even after 154 h of immersion in Hank’s solution, as shown in Figure 6d. Furthermore, the authors attributed the development of cracks on the surface of AZ31 Mg alloy and PEO to the dehydration in field-emission scanning electron microscopy (FESEM) and not due to the immersion in Hank’s solution. The Bode plots of the PEO coating and PEO/LDH composite coating are shown in Figure 6e,f. The impedance values |Z| at a frequency of 0.01 Hz of the AZ31 Mg alloy substrate, PEO coating, and PEO/LDH composite were found to be 478.03, 6.46 × 10^4^, and 3.45 × 10^6^ Ω·cm^2^. The Bode phase angle plots shown in Figure 6f showed that the PEO–LDH composite coating had the highest angle at the high-frequency region. As a result, significant improvements in the corrosion resistance of Mg alloy can be achieved by the combination of PEO and LDH coatings. Finally, it can be deduced that achieving a balance between the dissolution rate of the PEO coating and the growth rate of the LDH film by adjusting the pH and synthesis temperatures to higher and lower values, respectively, leading to the fabrication of a composite coating with superior corrosion performance.

#### 4.2.2. Modification of LDH Film Grown on PEO Coating

The correlation between the voltage applied during the PEO treatment of AZ31 Mg alloy and the growth of the LDHs film was examined by Zhang et al. [58]. The AZ31 Mg alloy samples were treated in an alkaline-aluminate electrolyte under different voltages, such as 150, 200, 250, 300, and 350 V for 10 min. Then, the LDH films were grown on the coated AZ31 Mg alloy samples via the hydrothermal method in a 0.1 M NaNO_3_ solution. The LDH growth was greatly affected by the dissolution rate of PEO coatings. The sample formed at 350 V showed a less number of LDH flakes which was attributed to the fact that MgAl_2_O_4_, which existed in higher amounts in the sample treated at 350 V, had lower solubility than the other phases, such as MgO and Al_2_O_3_. Although the thickness of the LDH film made on the PEO sample treated at 350 V was not higher than other samples, this sample exhibited the best corrosion resistance which was attributed to synergistic effects between LDH film and stable MgAl_2_O_4_.

The correlation between the hydrothermal treatment time and the corrosion performance of LDH–PEO composite coating formed on Mg–Li alloy was examined by Zhang et al. [90]. After the deposition of the coatings on Mg–Li alloy samples via PEO utilizing an electrolyte composed of NaOH, Na_2_SiO_3_·9H_2_O, and NaF, the coated samples were placed in an aqueous solution Al(NO_3_)_3_·9H_2_O for 12, 18, and 24 h at 363 K. NaOH was added to adjust the pH value to be 12.4. The microstructural results revealed that the typical porous morphology of PEO coatings tended to be sealed by LDH sheets where a dense LDH film with a lamellar structure was formed on the PEO-coated sample immersed in the hydrothermal solution for 24 h at 363 K. The compositional analysis of the coated samples approved the successful formation of LDH films on the surface of PEO coating which was mainly composed of MgO and Mg_2_SiO_4_. The authors attributed the formation of LDH film to the hydrothermal treatment which induces the partial dissolution of MgO which resulted in the deposition of Mg(OH)_2_ sheets, formed by the reaction of OH^−^ anions with the released Mg^2+^ cations, on the surface of the PEO coating. Based on the hydrogen evolution analysis and weight loss assessments in a 3.5 wt.% NaCl solution, it was established that the PEO coating had a higher corrosion rate than the PEO-LDH composite coating.

According to the recent study by Wang et al. [91], Mg–Al–Zn LDH films were successfully deposited on the PEO-coated AZ31 Mg alloy through a hydrothermal treatment in a solution of 0.05 M of Zn(NO_3_)_2_·6H_2_O without and with 0.01 and 0.05 M Al(NO_3_)_3_·9H_2_O solution. The results revealed that the MgO which is the main component of PEO coating tended to dissolve partially under the hydrothermal treatment, liberating Mg^2+^ ions, which combined with Al^3+^, Zn^2+^, and OH^−^ ions, forming Mg–Al–Zn LDH films, which in turn act as a sealing agent to the porous morphology of PEO coating. Per PDP and EIS measurements in a 3.5 wt.% NaCl solution, it was found that the formation of LDH film could greatly improve the corrosion resistance of PEO coating, regardless of the concentration of Al(NO_3_)_3_·9H_2_O. This result was ascribed to the filling effect and anion-exchange capacity of LDH films. Among different LDHs films, the protective properties of the LDH film made in a solution with a 0.05 M Al(NO_3_)_3_·9H_2_O were superior to other cases which were reflected by the lower value of *i_corr_* and higher impedance value in PDP and Bode plots, respectively.

Peng et al. [92] prepared LDH film on Mg alloy via PEO and hydrothermal treatment techniques in a solution of Al(NO_3_)_3_ containing different concentrations of Zn(NO_3_)_2_, such as 0, 10, 100, to 1000 µM. The samples obtained were denoted as Mg–Al LDH, Mg–Zn#1–Al–LDH, Mg–Zn#2–Al–LDH, and Mg–Zn#3–Al–LDH, respectively. Regardless of Zn(NO_3_)_2_ concentrations, the corrosion resistance of the AZ31 Mg alloy sample was improved by sealing the micropores of PEO coating with LDH flakes. Among all samples, Mg–Zn#3–Al–LDH sample exhibited the lowest corrosion rate as it had the lowest value of *i_corr_* and the highest value of polarization resistance (*R_p_*). Moreover, the Mg–Zn#3–Al–LDH sample showed an improved osteogenic activity and antibacterial ability, suggesting the potential usage of this sample in implant applications.

Wang et al. [93] prepared a superhydrophobic coating on the surface of PEO-coated AZ31 Mg alloy through the surface modification of LDH film by stearic acid (SA). Put simply, the Mg–Al LDH films were prepared by a hydrothermal process by immersion PEO-coated samples in a solution composed of Al(NO_3_)_3_ at 398 K for 6, 12, 18, and 24 h. The obtained samples were called PEO-LDH-6h, PEO-LDH-12h, PEO-LDH-18h, and PEO-LDH-24h, respectively. The PEO-LDH-24h sample was immersed in an ethanol solution containing 0.05 M of SA for 5 h at 333 K. The sample obtained was called PEO-LDH-24h/SA. The microstructural results revealed that the structural defects of PEO coating were gradually sealed by the growth of LDH flakes which tended to increase with an increase in the hydrothermal treatment time where a compact composite coating with a contact angle of 63.40° was produced on the surface of PEO-LDH-24h. Interestingly, the modification by SA led not only to more densification of the LDH film but also resulted in the formation of a superhydrophobic composite coating with a contact angle of 151.21°. The electrochemical measurements in a 3.5 wt.% NaCl solution revealed that the PEO-LDH-24h/SA sample had the highest corrosion resistance as compared to the substrate, PEO coating, and PEO-LDH-24h samples which were reflected by the highest impedance in the Bode plots. The improved corrosion resistance observed in the case of the PEO-LDH-24h sample was attributed to the synergism between hydrophobicity property and the self-healing ability of LDH film.

Zhang [57] prepared LDH films on the PEO-coated AZ31 Mg alloy via hydrothermal treatment. Briefly, The PEO-coated sample was first subjected to a conversion coating using a solution containing Ce(NO_3_)_3_ and H_2_O_2_ for 2 h at 323 K. This treatment led to the formation of a PEO–Ce sample. Afterward, the PEO–Ce sample was placed in 0.1 M NaNO_3_ at 398 K for 12 h under autoclave conditions which led to the formation of the PEO–Ce–LDH sample. The PEO–Ce–LDH samples were subjected to an anion-exchange reaction using an aqueous solution of phytic acid (PA) at 353 K for 1 h. The resultant sample was named PEO–Ce–LDH–P. The electrochemical measurements in a 3.5 wt.% NaCl solution indicated that the corrosion resistance of the PEO–Ce–LDH–P sample was superior to other samples which were linked to the synergistic effect between cerium and phosphate group from PA.

**Figure 7:**
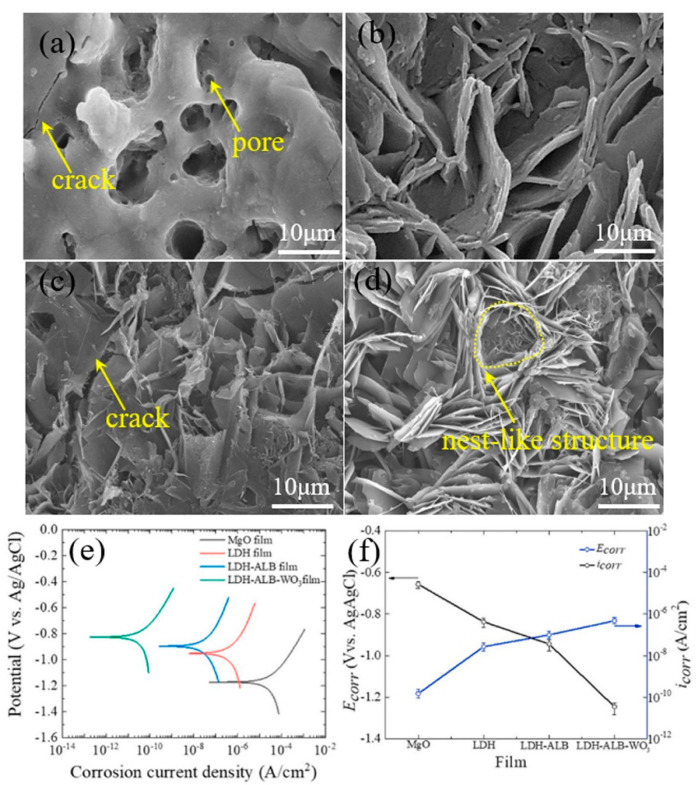
(**a**–**d**) SEM images showing the surface morphologies of MgO, LDH, LDH–ALB (albumin), and LDH–ALB–WO_3_ films, respectively. (**e**) Potentiodynamic polarization (PDP) curves of the obtained films in a 3.5 wt.% NaCl solution. (**f**) The variation of *E_corr_* and *i_corr_* the obtained films in a 3.5 wt.% NaCl solution. Reprinted with permission from ref. [94]. Copyright 2021 Elsevier.

Recently, Kaseem and Ko [94] modified the surface of LDHs film made on the PEO coating of AZ31 Mg alloy. Here, the LDHs films, prepared by co-precipitation method using a solution of Mg(NO_3_)_2_ and Al(NO_3_)_3_ at 423 K with a pH of 10.5, were modified by subsequent immersions in a solution containing albumin (ALB) molecules at 313 K for 1h which lead to the formation of LDH–ALB film. The LDH was also immersed in a solution comprising of albumin and WO_3_ nanoparticles at 323 K for 2h under continuous stirring which led to the formation of LDH–ALB–WO_3_ film. The results shown in Figure 7a–d, revealed that the deposition of ALB modified by WO_3_ nanoparticles could change the typical morphology of LDH film which led to the formation of a stable nest-like structure, hindering the penetration of corrosive anions through the film. From the PDP tests in a 3.5 wt.% NaCl solution shown in Figure 7e,f, it was found that the LDH–ALB–WO_3_ film had the lowest value of *i_corr_* and the most positive value of *E_corr_*, suggesting that this film could effectively trap the corrosive anions which were linked to the high stability of the albumin–WO_3_ composite. Furthermore, the authors attributed the enhanced corrosion resistance of LDH–ALB–WO_3_ film to the semiconducting properties of the composite film which led to potential barrier generation at the interface, preventing ease of corrosive anions motion towards the substrate.

Zhang and coworkers [95] prepared Mg–Al LDH films through the hydrothermal treatment of the PEO-coated AZ31 Mg alloy and reported that the porosity of the PEO coating was decreased after in situ formation of the LDHs, and thereby, showed a significantly long period of protection for the substrate. An Mg–Al–Co LDH film was successfully prepared on PEO-coated AZ31 Mg alloy by in situ hydrothermal growth method [96]. Briefly, the PEO coated samples were placed in a solution containing 0.05 M Al(NO_3_)_3_ and 0.02 M Co(NO_3_)_2_ for 24 h at 398 K under autoclave conditions. The pH solution was controlled to be 10.5 by adding ammonia. The results revealed that the surface of Mg–Al–Co LDH made on PEO coating was more compact and uniform than the PEO coating, indicating that the flakes of LDH film sealed the micro-pores and cracks in the PEO coating completely. The formation of Mg–Al–Co LDH film was attributed to the fact that the Mg^2+^ cations released by the gradual dissolution of the PEO coating tended to react with Al^3+^, Co^2+^, and OH^−^ ions in the hydrothermal solution. As a result of the successful formation of LDH film on the surface of PEO, the corrosion rate of the AZ31 Mg alloy substrate was significantly reduced as the *E_corr_* of the PEO coating increased from −1.47 to −1.39 V vs. SCE while the value of *i_corr_* of PEO coating was decreased by about two orders of magnitude upon the deposition of LDH film. Recently, Petrova et al. [97] studied the effects of chelating agents added into the reaction system on the growth of Mg–Al LDH films on PEO-coated AZ91 Mg alloy. It was reported that the inclusion of chelating agents would assist the formation of soluble metal complexes and thus the increase of amounts of soluble Al^3+^ and Mg^2+^ cations in the pH range of 9.6–10.3, which is suitable for LDH growth. On the other hand, the LDH films formed on PEO-coated pure Mg samples helped to improve the corrosion resistance and biocompatibility of Mg and to enhance osteogenic activity in vivo, indicating its promising potential for orthopedic applications [98].

### 4.3. Performance of LDH Films in Comparison with Other Post-Treatment Methods

As shown above, the production of LDHs film on the anodic films of Mg alloys helped to improve their corrosion resistance in corrosive media. However, a comparison between the effects of LDHs film in comparison with other surface modification methods on the corrosion performance of the anodic film of Mg alloys would be needed. Table 1 sums up the electrochemical parameters (*i_corr_* and *E_corr_*) obtained from PDP curves for the anodic films of Mg alloys (AZ31 and AZ91) post-treated via LDHs film and other surface modification methods. The conclusion can be drawn from Table 1 that the protective properties of the anodic films of Mg alloys can be improved by post-treatment methods, regardless of the type of post-treatment. However, the modification of the anodic films by LDH-based films would be more desirable and more effective than other methods due to the significant improvements in the corrosion resistance and long-term corrosion properties, feasible, and relatively eco-friendly procedures during the modification by LDH films.

## 5. Summary and Perspectives

Recent studies based on LDH/anodic film composites suggested that the fabrication of LDH films on the surface of anodic films produced on the Mg alloy by either anodizing or PEO would lead to fabricating functional materials for corrosion protection applications. Several processing methods have been developed to fabricate LDH/anodic film composites with a uniform and compact structure, which would be the main challenge before the success of commercialization. The in situ hydrothermal method has unique advantages in fabricating LDH/anodic film composites even though this method requires using autoclave and high temperatures. The formation of LDH film on the anodized samples could semi-seal or seal the structural defects, resulting in an improved corrosion property. However, the growth of the LDH film would be affected by several factors, such as the morphology of the anodic film, type of synthesis method, the composition of the hydrothermal solution as well as the processing parameters namely, pH, treatment time. The self-healing and ion-exchange capability of LDH film would enable it to release corrosion inhibitors and adsorb corrosive species when in contact with corrosive environments to restrain the corrosion progress. On the other hand, the LDH film can be fabricated also on the PEO coatings of Mg alloy; however, a balance between the dissolution rate of PEO coating and growth rate should be achieved to achieve the desired corrosion resistance. A superhydrophobic coating can be made through the surface modification of the LDH film which would further improve the protective properties of the obtained composites. LDH/PEO composite made on pure Mg can be used as promising implants in orthopedic applications.

Considering all the experimental results described in the present work, there is a clear potential for the use of LDH film as a final post-treatment to enable improvement in the performance of anodic films of Mg alloys. The key parameter for implementing the use of LDHs and derived materials is the choice of the appropriate synthesis process. Since the PEO coating has better performance than the anodized film, a better understanding of the PEO process and how the morphology and composition of the PEO coatings affect the formation of LDH film would be needed. This can be achieved by carrying out a systematic study on PEO coatings with different morphologies to have a better grasp on how to associate ideal barrier protection from the PEO coating itself and the sealing plus active corrosion protection from the “smart” LDH film. It is necessary to discover new eco-friendly corrosion inhibitors that can be intercalated into the LDH film by anion-exchange reactions since they are the main feature for active corrosion protection functionality. On the other hand, the stimulation of LDH film grown on the anodic films of Mg alloys would provide a better understanding of the functionality of these materials.

## Figures and Tables

**Table 1:** Summary of *i_corr_* and *E_corr_* obtained from the potentiodynamic polarization tests of the anodic films of Mg alloys post-treated by different methods. The units for *i_corr_* and *E_corr_* are μA·cm^−2^ and V, respectively, in the table.

Substrate	Solution/Electrode for Corrosion	Anodic Film		Post-Treatment	Anodic Film + Post-Treatment		Ref.
		*i_corr_*	*E_corr_*		*i_corr_*	*E_corr_*	
AZ31 Mg alloy	3.5 wt.% NaCl/SCE	3.89	−0.74	LDH-VO_3_ film	2.48 × 10^−1^	−0.40	[50]
		4.698	−1.48	Mg–Al LDH film	0.1178	−1.34	[63]
		2.31	−0.46	Ce-LDH-P film	0.05	−0.13	[57]
		^−^	-	LDH-Ce-V film	0.22	−0.173	[62]
		4.31 × 10^−2^	−1.474	LDH modified by stearic acid	1.78 × 10^−4^	−1.257	[93]
		0.413	−1.485	LDH + HT (0.05 M Al^3+^)	1.03 × 10^−3^	−1.350	[91]
		8.08	−0.59	LDH + HT (12 h)	0.79	−0.33	[55]
		10.33	−0.44	LDH+HT (0.032 M Al^3+^)	1.040	−0.372	[66]
		0.30	−1.58	high-intensity pulsed ion beam	4 × 10^−3^	−1.35	[99]
		2.58	−1.49	stearic acid coating	4.1 × 10^−2^	−1.37	[100]
		2.01	−1.42	polymer coating (polyproblene)	9.2 × 10^−4^	−1.47	[101]
		0.59	−1.61	HT	0.29	−1.48	[102]
		0.24	−1.46	polymethyltrimethoxy-silane sealing	2.86 × 10^−2^	−1.41	[103]
		0.13	−1.30	Ce-based sealing + Al(H_2_PO_4_)_3_ sealing	0.12	−1.29	[104]
	Hank’s solution/SCE	3.72	−1.64	LDH	5.69 × 10^−2^	−1.36	[88]
	3.5 wt.% NaCl/Ag/AgCl	2.26 × 10^−2^	−0.98	RF sputtering (Ni-Cr layer)	1.51 × 10^−3^	−0.61	[105]
		27.1	−1.183	LDH modified by albumin-WO_3_ composite	3.44 × 10^−5^	−0.832	[94]
	phosphate bufer saline/SCE	9.45	−1.22	LDH-HT	3.92	−1.2	[85]
	5 wt.% NaCl/Ag/AgCl	1.89	−1.37	electrophoretic coating	1.93 × 10^−2^	−1.28	[106]
	5 wt.% NaCl/Ag/AgCl	12.1	−1.75	inorganic sealing + CO_2_ solidifying	1.28 × 10^−3^	−1.65	[107]
	SBF/SCE	2.25	−1.56	self-assembly + thiolene photochemical reaction	1.67 × 10^−4^	−1.52	[108]
		3.06	−1.3	immersion in EDTACa/KH_2_PO_4_ solution at 353 K for 6 h	0.42	−0.76	[109]
		33	−1.39	ZrO_2_-sol gel sealing	2.40	−1.19	[110]
AZ91 Mg alloy	3.5 wt.% NaCl/SCE	1.27	−1.32	LDH modified by perfluoropolyether	2.04 × 10^−4^	−0.85	[86]
		0.39	−1.32	SiO_2_-ZrO_2_ sol-gel coating	1.57 × 10^−3^	−0.40	[76]
		1.60	−1.48	TiO_2_ sol-gel coating	0.08	−1.31	[14]
		0.40	−1.42	electrodeposition of silica + 8HQ	5.46 × 10^−3^	−1.38	[111]
		0.82	−1.46	Cyclic assembly	3.5 × 10^−2^	−1.36	[112]
		1.5	−1.87	La(NO_3_)_3_-based sealing	0.28	−1.65	[113]

HT = hydrothermal treatment, SBF = Simulated body fluid, SCE = Calomel electrode.

## Data Availability

The data presented in this study are available on request from the corresponding author.
